# Overexpression of Gremlin-1 in Patients with Loeys-Dietz Syndrome: Implications on Pathophysiology and Early Disease Detection

**DOI:** 10.1371/journal.pone.0104742

**Published:** 2014-08-12

**Authors:** Jasmin Wellbrock, Sara Sheikhzadeh, Leticia Oliveira-Ferrer, Hauke Stamm, Mathias Hillebrand, Britta Keyser, Marianne Klokow, Gabi Vohwinkel, Veronika Bonk, Benjamin Otto, Thomas Streichert, Stefan Balabanov, Christian Hagel, Meike Rybczynski, Frank Bentzien, Carsten Bokemeyer, Yskert von Kodolitsch, Walter Fiedler

**Affiliations:** 1 Hubertus Wald University Cancer Centre, Department of Oncology, Hematology and Bone Marrow Transplantation with section Pneumology, University Medical Centre Hamburg-Eppendorf, Hamburg, Germany; 2 Center of Cardiology and Cardiovascular Surgery, University Medical Centre Hamburg-Eppendorf, Hamburg, Germany; 3 Institute of Human Genetics, Hannover Medical School, Hannover, Germany; 4 Department of Clinical Chemistry/Central Laboratories, University Medical Centre Hamburg-Eppendorf, Hamburg, Germany; 5 Division of Hematology, University Hospital Zurich, Zurich, Switzerland; 6 Institute for Neuropathology, University Medical Centre Hamburg-Eppendorf, Hamburg, Germany; 7 Department of Transfusion Medicine, University Medical Centre Hamburg-Eppendorf, Hamburg, Germany; Children's Hospital Los Angeles, United States of America

## Abstract

**Backgrounds:**

The Loeys-Dietz syndrome (LDS) is an inherited connective tissue disorder caused by mutations in the transforming growth factor β (TGF-β) receptors *TGFBR1* or *TGFBR2*. Most patients with LDS develop severe aortic aneurysms resulting in early need of surgical intervention. In order to gain further insight into the pathophysiology of the disorder, we investigated circulating outgrowth endothelial cells (OEC) from the peripheral blood of LDS patients from a cohort of 23 patients including 6 patients with novel TGF-β receptor mutations.

**Methods and Results:**

We performed gene expression profiling of OECs using microarray analysis followed by quantitative PCR for verification of gene expression. Compared to OECs of age- and sex-matched healthy controls, OECs isolated from three LDS patients displayed altered expression of several genes belonging to the TGF-β pathway, especially those affecting bone morphogenic protein (BMP) signalling including *BMP2*, *BMP4* and *BMPR1A*. Gene expression of BMP antagonist Gremlin-1 (*GREM1*) showed the most prominent up-regulation. This increase was confirmed at the protein level by immunoblotting of LDS-OECs. In immunohistochemistry, abundant Gremlin-1 protein expression could be verified in endothelial cells as well as smooth muscle cells within the arterial media. Furthermore, Gremlin-1 plasma levels of LDS patients were significantly elevated compared to healthy control subjects.

**Conclusions:**

These findings open new avenues in the understanding of the pathogenesis of Loeys-Dietz syndrome and the development of new diagnostic serological methods for early disease detection.

## Background

The Loeys-Dietz syndrome (LDS) is an inherited autosomal dominant connective tissue disorder described first in 2005 by Bart Loeys and Harry Dietz [1]. Most common characteristics of LDS patients are 1) craniofacial features such as hypertelorism, cleft palate with bifid uvula, 2) skeletal manifestations including joint laxity, scoliosis and arachnodactyly, 3) cutaneous findings such as translucent skin or easy bruising and 4) vascular manifestations affecting the aorta and other arterial branches resulting in early need of surgical intervention [1], [2].

The Loeys-Dietz syndrome is caused by a mutation in the transforming growth factor β (TGF-β) type II receptor *TGFBR2* or type I receptor *TGFBR1*. More than 50 different mutations in *TGFBR2* or *TGFBR1* have been described in LDS patients. The great majority of those mutations represent missense mutations which are located within the kinase domain of the receptor probably resulting in impaired receptor signalling [1]–[3]. Recently, mutations in the gene for *TGFB2* and the TGF-β pathway downstream mediator *SMAD3* have also been associated with the pathogenesis of Loeys-Dietz syndrome [4]–[6].

The TGF-β superfamily consists of several isoforms of TGF-β, activin and bone morphogenic proteins (BMP). Signalling is mediated through two related transmembrane type I and type II serine/threonine kinase receptors, which form heteromeric complexes upon ligand binding and propagate the downstream signal by phosphorylation of intracellular SMAD proteins which transduce the signal to the nucleus [7].

The vascular wall is a complex construct composed of different cell types including the inner layer of endothelial cells (EC), surrounded by smooth muscle cells (SMC) within the media and finally the adventitia composed of fibroblasts [8]. For function of the vascular wall, interdependency between endothelial cells and mural cells is required. Communication can take place via direct cellular or via paracrine interactions induced by secretion of molecules such as platelet-derived growth factor [9]. With regard to the endothelium's crucial role for the maintenance of the integrity of the vascular wall, we decided to concentrate our studies on endothelial cells to elucidate the pathogenesis of Loeys-Dietz syndrome. A population of circulating endothelial cells, so called outgrowth endothelial cells (OEC), can be easily isolated from peripheral blood for this purpose [10]. Moreover, OECs isolated from patients with hereditary haemorrhagic teleangiectasia (HHT) carrying mutations in TGF-β receptors *ACVRL1* (ALK-1, activin receptor-like kinase 1) or *ENG* (endoglin) displayed abnormalities comparable to the vascular lesions observed in HHT patients [11].

Therefore, we isolated outgrowth endothelial cells from the peripheral blood of LDS patients and healthy donors and performed gene expression profiling in order to study aberrant gene regulation caused by mutated TGF-β receptors. The aim of our study was to identify candidate genes contributing to the disease pattern of Loeys-Dietz syndrome.

## Methods

### Generation of outgrowth endothelial cells

The investigation conforms with the principles outlined in the Declaration of Helsinki. Written informed consent was obtained from individuals participating in the study after the study had been approved by the local ethical committee [PV3893, Ärztekammer Hamburg]. Mononuclear cells (MNC) were isolated from peripheral blood of LDS patients and healthy donors, plated in collagen-coated 12-well tissue culture plates and cultured in endothelial growth medium (Lonza, Walkersville, MD, USA) supplemented with 10% fetal bovine serum (Invitrogen, Carlsbad, CA, USA). In order to remove non-adherent cells and debris, cultures were rinsed daily with fresh medium for one week followed by medium replacement every other day. On day 30, cultures were screened for outgrowth of endothelial colonies. Endothelial character of OEC clones was confirmed in PCR analysis and flow cytometry based on expression of a panel of endothelial-specific markers including CD31, CD144 and vascular endothelial growth factor receptors and non-expression of haematological markers CD45 and CD14 (see methods and table S1 in file S1).

### Mutation analysis and prediction of the functional impact of nucleotide or amino acid substitutions

DNA was extracted from EDTA-blood using standard procedures. The entire coding sequence of *TGFBR1* (NM_004612.2) and *TGFBR2* (NM_003242.5) was sequenced as well as the 20 bases of the flanking intronic sequences. The amplified PCR products were sequenced and analysed with the following bioinformatics tools for prediction of impact on protein function: Mutation Taster (http://www.mutationtaster.org/), PMut (http://mmb2.pcb.ub.es:8080/PMut/), PolyPhen (http://genetics.bwh.harvard.edu/pph/), PolyPhen2 (http://genetics.bwh.harvard.edu/pph2/). Presumptive splice site changes caused by silent or intronic mutations were analysed with the Human Splicing Finder tool, [12] Berkeley Drosophila Genome Project “Splice Site Prediction” (http://www.fruitfly.org/seq_tools/splice.html) and NetGene2 Server (http://www.cbs.dtu.dk/services/NetGene2/). The non-mutation carrying chromosomes of 400 Marfan and Loeys-Dietz syndrome patients were used as control chromosomes.

### RNA isolation and microarray analysis

RNA was extracted using RNeasy Mini Kit (including RNase-free DNase Set, Qiagen, Hilden, Germany). For microarray analysis, quality and concentration of isolated RNA was determined using the Agilent RNA 6000 Nano Kit (Agilent Technologies, Loveland, CO). Procedures for cDNA synthesis, labelling and hybridization were carried out according to 3′ IVT Express Kit and Hybridization, Wash and Stain Kit (Affymetrix, Santa Clara, CA) using 100 ng total RNA. All experiments were performed using Human GeneChip U133 Plus 2.0 Array (Affymetrix). Microarrays were scanned with the GeneChip Scanner 3000 7G. The signals were processed with GeneChip Operating Software (version 1.4, Affymetrix). Signal quality control and data normalization via gcrma procedure was performed using the webserver www.arrayanalysis.org. Differentially expressed genes were determined by filtering out genes that were increased or decreased at least 1.74 fold (Signal Log Ratio ≥0.8) in each sample pair and exhibited a permutation p-value below 0.05. Gene expression data are available at GEO Accession No. GSE38961.

### cDNA synthesis and Real-Time quantitative PCR

RNA was reverse transcribed using the Ready-To-Go You-Prime First-Strand Beads (GE Healthcare, Fairfield, CT) and Random Primers (Invitrogen). Primers were designed with Primer 3 software (Whitehead Institute for Biomedical Research, Boston, MA). Quantitative Real-Time PCR analysis was carried out on the capillary-based Light Cycler (Roche, Basel, Switzerland) using the FAST Start DNAMaster Sybr Green Kit (Roche). Relative expression of cDNA of the target gene in comparison to a reference gene was calculated using a mathematical model proposed by Pfaffl [13]. Samples were analysed in duplicate and averaged. Calculated cDNA amounts of the target genes were normalized to the reference gene glyceraldehyde-3-phosphate dehydrogenase (*GAPDH*). All data are represented as ratio of the target gene/*GAPDH*. Primers are shown in table S2 in file S1.

### Immunohistochemistry

For immunohistochemical labelling, formalin-fixed paraffin-embedded aortic tissue sections of LDS patients or healthy donors were pre-treated in citrate buffer and incubated with antibodies against Gremlin-1 (bs-1475R, Bioss, Woburn, MA) in an automated stainer (Ventana Medical Systems, Tucson, AZ) according to a standard protocol (CC1st). For double labelling with Gremlin-1 and muscle actin (ENZ-30931, Enzo Life Sciences GmbH, Loerrach, Germany) or Gremlin-1 and CD34 (M7165, Dako, Hamburg, Germany), incubation with Gremlin-1 antibodies was followed by a short denaturing step and incubation with the second antibody. Bound antibodies were detected by the peroxidase method using diaminobenzidine as chromogen (760–500, Ultraview DAB, Ventana). For double labelling studies, bound Gremlin-1 antibodies were visualized with DAB as described above and expression of muscle actin or CD34 was demonstrated by alkaline phosphastase linked secondary antibodies using fast red as chromogen (760–501, Ultraview Universal Detection Kit, Ventana).

### Immunoblotting

Protein extracts were prepared with RIPA lysis buffer solution (Sigma-Aldrich, St. Louis, MO) supplemented with 1× protease inhibitor cocktail (Roche) and 1 mM sodium orthovanadate. Protein lysates were boiled for 5 Min in SDS-sample buffer before being applied into a 4–20% SDS-PAGE (Thermo Fisher Scientific, Rockford, IL). After electrotransfer to nitrocellulose membranes (Schleicher & Schuell, Dassel, Germany) and blocking in TBS-T buffer containing 5% non-fat milk for 1 h, blots were incubated with Gremlin-1 primary antibody (Santa Cruz, Santa Cruz, CA) overnight. The subsequent incubation with the peroxidase-conjugated secondary antibody was followed by detection using ECL Western blotting detection reagents (GE Healthcare) and the FusionSL 4 3500 WL detection system (Vilber Lourmat, Sud Torcy, France). Membranes were incubated with Restore Western Blot Stripping Buffer (Thermo Fisher Scientific) followed by incubation with an α-Tubulin antibody (Sigma-Aldrich) as reference protein. Quantification of protein amount was determined using Bio-1D software (Vilber Lourmat).

### Collection of human plasma samples and anti-human Gremlin-1 ELISA analysis

For analysis of Gremlin-1 plasma levels, peripheral blood samples of LDS patients and healthy donors were collected and centrifuged for 10 min at 2,000 g in order to separate the plasma from the blood cells. Gremlin-1 protein levels were measured using the enzyme-linked immunosorbent assay (ELISA) Kit for Gremlin-1 (Uscn Life Science Inc., Missouri City, TX) following the instructor's manual. Absorbance of color change was quantified with the Sunrise ELISA plate reader and Magellan software (Tecan, Maennedorf, Switzerland).

### Statistical analysis

All statistical analyses were performed with SPSS 16 (SPSS Inc, Chicago, IL). OEC frequency was analysed using the Mann-Whitney-U test. Differences in Gremlin-1 plasma levels between LDS patients and healthy controls or between gender were accessed by Welch's t-test. Correlation of Gremlin-1 plasma levels with age was analysed by Pearson's correlation. P-value≤0.05 was considered as statistically significant. For the microarray data, a permutation procedure was performed to obtain adjusted p-values. Differentially expressed genes were identified by filtering out genes with a permutation p-value below 0.05 and a minimum absolute signal-log-ratio of 0.8 in each of the three sample pairs.

## Results

### LDS patients and novel mutations in TGFBR2 and TGFBR1

By evaluation of clinical presentation and mutation analysis of *TGFBR1* and *TGFBR2*, 23 patients were diagnosed with Loeys-Dietz syndrome at the University Medical Centre Hamburg-Eppendorf. These 23 patients belong to thirteen families. Sixteen patients carry a heterozygous mutation in the *TGFBR2* gene and seven patients harbour a *TGFBR1* mutation. We identified four novel mutations in *TGFBR2* and two in *TGFBR1* whereas the other mutations have already been described before (table 1) [1]–[3]. Two of the novel *TGFBR2* mutations are located within the kinase domain, both of them representing missense mutations (p.N384K and p.A414T). Analysis with bioinformatic tools Mutation Taster, PMut, Polyphen and PolyPhen2 predicted both mutations *TGFBR2* p.N384K and p.A414T to be “disease causing” (data not shown). Furthermore, a silent mutation located in the region between the transmembrane and kinase domain of *TGFBR2* was found (p.A232A). In addition, one patient carried a nucleotide substitution within intron 1 of the *TGFBR2* gene (c.94+7G>C). In order to determine if these nucleotide changes might lead to alternative splicing sites, data analysis was performed for the silent mutation and the intronic variant within intron 1 using bioinformatic tools. Nucleotide change c.94+7G>C in *TGFBR2* was predicted to cause an alternative splicing site in one of the analyses whereas no RNA splicing variants were predicted for *TGFBR2* p.A232A (data not shown).

**Table 1:** Heterozygous *TGFBR2* and *TGFBR1* mutations identified in LDS patients.

Gene	Location	Nucleotide change	Amino acid change	Type	Affected individuals	Mutation referenced in
*TGFBR2*	Intron 1	c.94+7G>C	Not known	Nucleotide substitution	1	Novel mutation
*TGFBR2*	Exon 4	c.696C>T	p.A232A	Silent mutation	1	Novel mutation
*TGFBR2*	Exon 4	c.1152T>G	p.N384K	Missense	3	Novel mutation
*TGFBR2*	Exon 4	c.1159G>A	p.V387M	Missense	1	Stheneur *et al.*, Matyas *et al.* [3], [34]
*TGFBR2*	Exon 4	c.1167C>T	p.N389N	Silent mutation	4	Stheneur *et al.* [3]
*TGFBR2*	Exon 4	c.1240G>A	p.A414T	Missense	1	Novel mutation
*TGFBR2*	Exon 7	c.1583G>A	p.R528H	Missense	1	Loeys *et al.*, Stheneur *et al.* [1], [3]
*TGFBR2*	Exon 7	c.1609C>T	p.R537C	Missense	4*	Loeys *et al.,* Stheneur *et al.* [2], [3]
*TGFBR1*	Intron 1	c.97+25_+39dup15	Not known	Duplication	1	Novel mutation
*TGFBR1*	Exon 4	c.721T>C	p.S241P	Missense	1	Novel mutation
*TGFBR1*	Exon 9	c.1433A>G	p.N478S	Missense	1	Loeys *et al.* [2]
*TGFBR1*	Exon 9	c.1460G>A	p.R487Q	Missense	4	Loeys *et al.,* Matyas *et al.* [2], [34]

*representing two unrelated families.

One of the novel *TGFBR1* mutations occurred at amino acid position 241 in exon 4 leading to substitution of serine with proline (p.S241P) which was predicted to be “disease causing” using bioinformatic tools (data not shown). The second novel *TGFBR1* mutation represented a duplication of 15 base pairs within intron 1 (c.97+25_+39dup15). The altered sequence was subjected to splice site analysis but no indications of alternative splice sites could be found (data not shown). All nucleotide changes detected within our cohort of LDS patients are summarized in table 1.

### Generation of outgrowth endothelial cells from LDS patients

Peripheral blood from only nine patients with Loeys-Dietz syndrome was available for generation of outgrowth endothelial cells. Six patients had a mutation in the TGF-β type II receptor *TGFBR2* and 3 patients carried a mutated type I receptor *TGFBR1*. Patient's characteristics are shown in table 2. The mean number of mononuclear cells obtained from the patients was 3.6×10^7^±1.8×10^7^. After a cultivation period of 30 days, seven OEC clones could be isolated from four patients resulting in a frequency of 1.94 clones per 10^8^ mononuclear cells. This compares to OECs isolated from healthy donors occurring with a frequency of 0.75 clones per 10^8^ mononuclear cells (n = 500; p = 0.257 by Mann-Whitney-U test). To confirm their endothelial character, OEC clones were analysed by flow cytometry between passages 3–6. Expression of endothelial-specific markers such as CD31 or CD144 and non-expression of haematopoietic markers CD45 and CD14 was comparable in LDS-OECs and OECs isolated from healthy donors (table S1 in file S1).

**Table 2:** LDS patients analysed for OEC generation.

Patient	Sex	Age	Mutation	LDS-OEC*
LDS1/LDS9	Female	54	*TGFBR2* p.R537C	2
LDS2	Male	24	*TGFBR2* p.R537C	0
LDS3	Male	50	*TGFBR2* p.A414T	0
LDS4/LDS10/LDS13	Male	26	*TGFBR1* duplication	3
LDS5	Male	27	*TGFBR2* p.R537C	1
LDS6	Male	55	*TGFBR2* p.N389N	0
LDS7	Male	64	*TGFBR1* p.R487Q	0
LDS8	Male	26	*TGFBR2* p.R537C	0
LDS11/LDS12	Female	28	*TGFBR1* p.R487Q	1

* number of generated OEC clones.

### Gene profiling of outgrowth endothelial cells from LDS patients

Due to insufficient *in vitro* proliferation capacity, not all OEC clones were available for microarray analysis. OEC clones from patients LDS1, LDS5 and LDS11 were used for gene profiling. LDS1 and LDS5 both carried the p.R537C mutation in the *TGFBR2* gene whereas LDS11 harboured the *TGFBR1* mutation p.R487Q. Detailed patient characteristics are provided in table 3. OEC clones from sex- and age-matched healthy donors served as reference. Furthermore, all OEC clones were harvested at passage 4-5 when nearly reaching confluence to reduce culture-induced variability.

**Table 3:** Patient characteristics of LDS1, LDS5 and LDS11.

LDS patient	LDS1	LDS5	LDS11
**Sex**	Female	Male	Female
**Age**	54 years	27 years	28 years
**Mutation**	*TGFBR2* p.R537C	*TGFBR2* p.R537C	*TGFBR1* p.R487Q
**Family history for sudden cardiac death**	Yes	No	Yes
**Aortic root diameter (Δ95th)^A^**	4.4 cm (+0.7 cm)	3.0 cm (−0.8 cm)	4.0 cm (+0.5 cm)
**Further aneurysms**	Yes	No	No
**Arterial tortuosity**	Yes	No	No
**Mitral valve prolapse**	Yes	No	Yes
**Dural ectasia**	Yes	No	Yes
**Cranio-facial features**	Bifid uvula, hypertelorism	Bifid uvula	Bifid uvula, hypertelorism
**Skeletal features**	Pectus carinatum	Protusio acetabuli, pes planus, hypermobile joints	Scoliosis, pectus excavatum, protusio acetabuli, pes planus, hypermobile joints
**Skin features**	No	No	No

* Δ95th identifies the difference of diameters obtained in study patients at baseline minus diameter (cm) at 95th percentile as assessed according to Biaggi *et al.* [35].

Expression ratios were considered as altered if the signal log ratio was above 0.8 (increased expression) and below −0.8 (decreased expression). In addition, only genes with consistently altered expression in all three analysed OEC clones were used for further analysis resulting in 163 genes with increased and 210 genes with decreased expression. In order to identify affected signalling cascades, the set of involved genes was analysed using the Ingenuity Pathways Analysis algorithm (IPA Build 308606M, Ingenuity Systems, www.ingenuity.com). Data analysis ranked the topics “Cardiovascular Disease” and “Haematological System and Cardiovascular System Development and Function” within the top five of affected biological functions and networks reflecting the actual type of genetic disorder (data not shown).

In all three LDS-OEC clones, gene expression of BMP antagonist Gremlin-1 (*GREM1*, also known as Drm) showed the most prominent up-regulation. Verification of expression using quantitative RT-PCR analysis and normalization to reference gene glyceraldehyde-3-phosphate dehydrogenase (*GAPDH*) revealed a 1,136-, 164- and 22,145-fold higher expression in LDS-OECs compared to healthy controls (table 4).

**Table 4:** Members of the TGF-β superfamily with altered mRNA expression levels in LDS-OECs compared to healthy controls.

*GREM1*			
	Signal log ratio*	Fold Change^†^	Relative expression^‡^
**LDS1/BC248**	6.3	80	1136
**LDS5/BC14**	9.3	617	164
**LDS11/BC401**	12.5	5873	22145

*BMPR1A*			
	Signal log ratio*	Fold Change^†^	Relative expression^‡^
**LDS1/BC248**	5.2	35	2919
**LDS5/BC14**	1.5	2.7	15
**LDS11/BC401**	6.4	82	704

*LTBP1*			
	Signal log ratio*	Fold Change^†^	Relative expression^‡^
**LDS1/BC248**	0.9	1.8	1.9
**LDS5/BC14**	2.0	3.9	1.9
**LDS11/BC401**	2.5	5.6	4.8

*BMP2*			
	Signal log ratio*	Fold Change^†^	Relative expression^‡^
**LDS1/BC248**	−1.8	−3.4	0.7
**LDS5/BC14**	−2.6	−6.0	0.2
**LDS11/BC401**	−1.0	−1.9	0.4

*BMP4*			
	Signal log ratio*	Fold Change^†^	Relative expression^‡^
**LDS1/BC248**	−1.9	−3.6	0.6
**LDS5/BC14**	−1.8	−3.5	0.1
**LDS11/BC401**	−9.2	−605	<0,1

* signal log ratio of LDS-OEC compared to healthy control, determined in microarray analysis; ^†^ expression fold change of LDS-OEC compared to healthy control, converted from microarray data; ^‡^ relative gene expression in LDS-OEC compared to healthy control, determined in quantitative PCR analysis and normalized to *GAPDH* expression.

In addition to *GREM1*, several other genes belonging to the TGF-β superfamily displayed altered expression levels in LDS-OECs, especially those affecting bone morphogenic protein signalling. Expression of BMP type I receptor *BMPR1A* was increased in all three LDS-OEC clones, whereas *BMPR1A* ligands *BMP2* and *BMP4* showed decreased mRNA expression. Furthermore, expression of the latent TGF-β binding protein 1 (*LTBP1*) was increased in LDS-OECs (table 4). In support of our findings, gene expression data were analysed calculating a permutation-derived adjusted p-value. This analysis revealed that all genes we have chosen for our analysis, namely *GREM1, BMP2, BMP4, BMPR1A* and *LTBP1*, yielded a permutation p-value of 0.0 (table S3 in file S2).

### Increased Gremlin-1 protein expression in LDS-OECs

**Figure 1:**
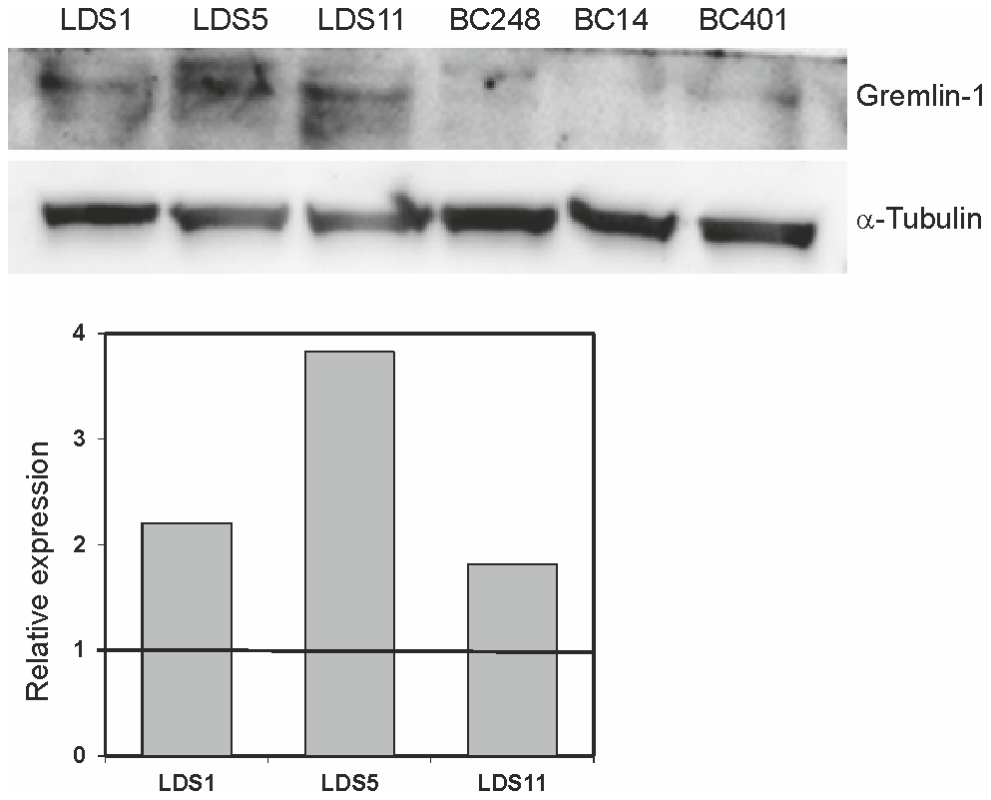
Elevated Gremlin-1 protein expression in LDS-OECs. The Gremlin-1 protein expression in LDS-OECs was compared to OECs isolated from sex- and age-matched healthy donors. Immunoblotting followed by quantification revealed that the Gremlin-1 protein amount was increased in all three LDS-OEC clones compared to their respective control.

Western blot analysis of LDS-OECs and sex- and age-matched control OECs confirmed a higher Gremlin-1 protein expression in LDS-OECs by 2.2-, 3.8- and 1.8-fold in LDS1, LDS5 and LDS11 respectively, compared to control OECs (figure 1).

### Gremlin-1 expression in aortic tissue of LDS patients

**Figure 2:**
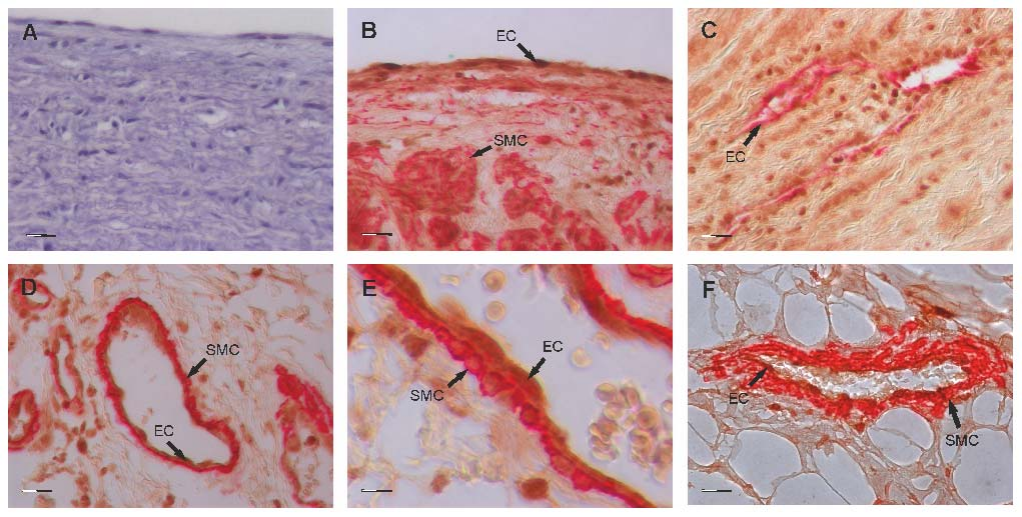
Gremlin-1 expression on aortic tissue of LDS patients. Paraffin-embedded aortic tissue specimen of LDS patients (n = 3) were double stained with anti-Gremlin-1 (brown staining) and anti-CD34 (red staining; C) or anti-smooth muscle actin (red staining; B, D–F). Endothelial cells throughout the vessel wall showed expression for Gremlin-1 including ECs of the intima (B) and of vessels within the media (C) and the adventitia (D and in more detail in E). Gremlin-1 positive staining was also observed on smooth muscle cells of the media (B, C) as well as on vessel surrounding smooth muscle cells in the adventitial layer (D, E). In aortic tissue specimen of healthy controls (n = 3), a similar staining pattern without gross differences of staining intensity was observed as shown for a small vessel within the adventitia (F). In A, an isotype control instead of primary antibody was used revealing the specificity of the staining (EC = endothelial cell, SMC = smooth muscle cell; magnification A–D, F: 400×; E: 1000×; scale bar represents 20 µm in A–D and F and 8 µm in E).

Aortic tissue specimen of LDS patients which had experienced aortic root replacement (n = 3; 2×TGFBR1 p.R487Q and 1×TGFBR2 p.R537C, the latter was corresponding to LDS1) and healthy donors (n = 3) were analysed for protein expression of Gremlin-1 in immunohistochemistry. Endothelial cells throughout the vessel wall layers stained positively for Gremlin-1 including ECs of the intimal layer as well as ECs of the vessels within the media and adventitia (figure 2). Furthermore, medial and vessel-surrounding smooth muscle cells were positive for Gremlin-1 (figure 2). Immunohistochemically, no gross differences of staining intensity or pattern between LDS patients and healthy controls could be observed probably because expression levels are difficult to quantify by immunohistochemistry (figure 2).

### Elevated Gremlin-1 plasma levels in LDS patients

**Figure 3:**
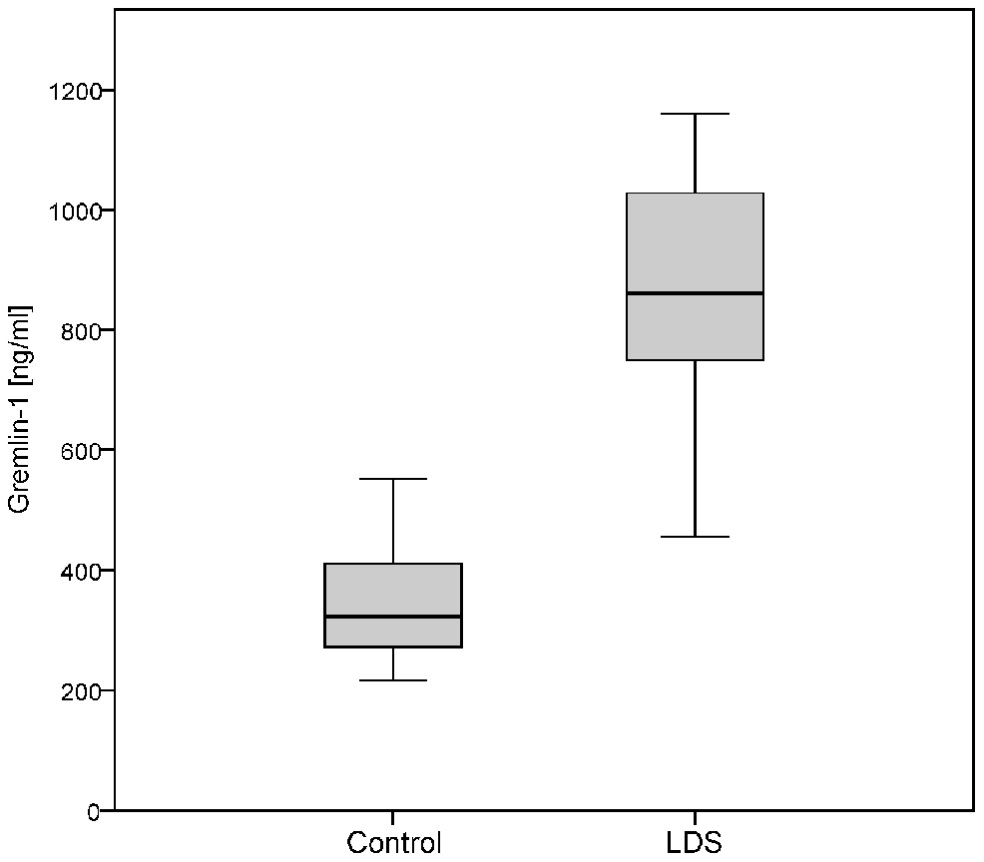
Gremlin-1 plasma levels are significantly increased in LDS patients. Gremlin-1 plasma levels of LDS patients (n = 9) were analysed in an enzyme-linked immunosorbent assay. Compared to healthy donors (n = 15), mean plasma levels of Gremlin-1 were 2.5-fold increased in LDS patients (Welch's t-test p<0.001). Box plots show the median (central horizontal line), the 25th to the 75th percentile (box) and the range (whiskers).

Since Gremlin-1 was the gene with the most prominent increase in LDS-OECs, we wondered if an elevated Gremlin-1 protein expression could also be detected systemically. Therefore, we analysed plasma samples from LDS patients and healthy controls (n = 9 and n = 15, respectively) using a commercial human Gremlin-1 ELISA. The mean Gremlin-1 plasma level of LDS patients was 2.5-fold increased with 858±228 ng/ml, compared to those of healthy donors with 349±107 ng/ml (Welch's t-test p<0.001; figure 3). The differences of Gremlin-1 levels were independent of donor's age (Pearson-Rho = 0.125, p = 0.560) or sex (t-test p = 0.514).

## Discussion

Twenty-three LDS patients belonging to 13 different families who are cared for at the University Medical Centre Hamburg-Eppendorf were molecularly characterised. In addition to mutations previously reported in LDS patients [2], [3], we identified six novel mutations. Although the great majority of mutations in Loeys-Dietz syndrome are missense mutations located within the kinase domain of *TGFBR2* or *TGFBR1*, only three of our six novel mutations fit within this category: p.N384K and p.A414T in *TGFBR2* and p.S241P in *TGFBR1* which were all predicted to be probably disease causing in bioinformatic analysis.

Mutation p.A232A in *TGFBR2* represented a synonymous mutation not leading to an amino acid change. This mutation could not be detected in any of the 400 control chromosomes therefore probably not representing a single nucleotide polymorphism. Furthermore, we identified two novel mutations which were located in non-coding DNA regions: a nucleotide substitution within intron 1 of the *TGFBR2* gene (c.94+7G>C) and a duplication of 15 base pairs within intron 1 of the *TGFBR1* gene (c.97+25_+39dup15). Nevertheless, mutations not affecting the protein sequence may account for disease manifestations in LDS as they do in cystic fibrosis, infantile spinal muscular atrophy or Crohn's disease [14].

In order to investigate whether endothelial cells contribute to the pathophysiology of Loeys-Dietz syndrome, we isolated outgrowth endothelial cells from nine LDS patients who volunteered to donate peripheral blood for research purposes. Due to the low frequency of OEC clones and insufficient proliferation capacity *in vitro*, the gene expression profile of only three LDS-OEC clones could be compared to age- and sex-matched healthy controls in microarray analysis. Although LDS11 carried a mutation in the type I receptor *TGFBR1* whereas LDS1 and LDS5 harboured the mutation p.R537C in type II receptor *TGFBR2*, more than 250 genes could be identified that displayed significant alterations in gene expression in all three analysed LDS-OEC clones. Genes with altered expression included several members of the TGF-β superfamily. Strikingly, most of them affected bone morphogenic protein signalling, namely *BMPR1A, BMP2, BMP4* and *GREM1* which displayed the most prominent up-regulation.

Recently, several studies revealed a direct link between TGF-β and Gremlin-1 signalling. Interestingly, although the majority of TGF-β receptor mutations in LDS patients results in non-functional receptor kinase activity, increased phosphorylation levels of the TGF-β downstream mediator proteins SMAD2 and SMAD3 have been observed in the aortic tissue of LDS patients [1], [15]. In concordance with these observations, the expression of a kinase-deficient TGFBR2 variant in a transgenic mouse model resulted in TGF-β overactivity including increased SMAD2/3 phosphorylation and induced development of fibrosis [16]. Several recently published studies could directly link TGF-β-induced phosphorylation of SMAD2/3 to increased Gremlin-1 expression [17]–[19]. Hence, elevated Gremlin-1 expression levels might represent a direct consequence of the dysregulated TGF-β signalling in LDS-OECs.

The drastic increase of Gremlin-1 was not only confirmed by Western Blotting of LDS-OECs, we also observed significantly elevated Gremlin-1 plasma levels in LDS patients compared to healthy subjects, suggesting that up-regulation of Gremlin-1 was a systemic phenomenon.

Although larger studies are needed to confirm increased plasma levels of Gremlin-1 in LDS, our observation may have many practical implications. Determination of Gremlin-1 concentration in peripheral blood may serve as a quick screening assay in patients with vascular abnormalities and direct more detailed molecular analysis. Since the median life expectancy in a large study of LDS was only 26 years and many patients with LDS are unrecognized, such a screening assay would permit early disease detection and timely surgical intervention [2]. Furthermore, a future molecularly targeted therapy may be followed by serial determination of Gremlin-1 plasma levels.

Gremlin-1 is a highly conserved 184 amino acid, secreted glycoprotein protein belonging to the cysteine knot superfamily. Gremlin-1 can bind and therefore antagonize bone morphogenic proteins, namely BMP2, BMP4 and BMP7 [20], [21]. During embryogenesis, Gremlin-1 is indispensable since mice with a homozygous deletion of the Gremlin-1 gene die shortly after birth due to complete renal agenesis and lung septation defects [22]. Gremlin-1 plays a role in several vascular diseases such as diabetic nephropathy or retinopathy [23], [24].

Contribution of Gremlin-1 to the pathogenesis of Loeys-Dietz syndrome may be explained by effects on vascular cells [25]. By binding to BMP2 and BMP4, Gremlin-1 should antagonize the proangiogenic BMP effects on endothelial cells therefore exhibiting antiangiogenic properties. But to the contrary, recently published data revealed that Gremlin-1 can mediate strong angiogenic effects via direct binding to the vascular endothelial growth factor receptor 2 (VEGFR2). Proangiogenic properties such as *in vitro* induction of proliferation, migration and vascular sprouting of endothelial cells were comparable to those achieved upon stimulation with vascular endothelial growth factor A (VEGF-A) [26], [27]. Hence in endothelial cells, Gremlin's proangiogenic effects via binding to VEGFR2 seem to predominate the antagonizing effects on bone morphogenic proteins. Therefore we suppose that Gremlin-1 mediates predominantly proangiogenic properties in LDS-OECs. This presumption is supported by the fact that expression of proangiogenic BMP2 and BMP4 is consequentially down-regulated in LDS-OECs.

Since Gremlin-1 is a secreted factor, it might not serve as an autocrine regulator of endothelial cells but may also mediate paracrine effects on other cell types. This assumption is strengthened by the fact that the Gremlin-1 plasma levels were significantly increased in LDS patients compared to healthy controls. The aortic media of LDS patients is characterized by a disorganized wall and diffuse medial degeneration with marked excess of collagen and loss of elastic fiber architecture [1], [15]. Gremlin-1 might account for some of these characteristics since it has been described to play a role in extracellular matrix modulation [24], [28]. In a diabetic nephropathy mouse model, a first therapeutic approach with Gremlin-1 siRNA was conducted. Inhibition of Gremlin-1 decelerated diabetic nephropathy through decrease of proteinuria, renal collagen accumulation and renal cell proliferation and apoptosis [29].

Maciel *et al.* investigated whether Gremlin-1 had an impact on vascular smooth muscle cells. SMCs overexpressing Gremlin-1 showed markedly increased proliferation and migration capacities compared to empty-vector transfected cells. In contrast, both proliferation and migration were reduced after gene silencing with shRNA against Gremlin-1 mRNA [30]. Our immunohistological data support these findings since Gremlin-1 expression was mainly observed in the endothelial layer of the intima or in small vessels in the adventitia and in smooth muscle cells of the media.

Recently, Cahill *et al.* reported that Gremlin-1 plays a key role in pulmonary arterial hypertension (PAH) [31]. The majority of patients with the heritable form of PAH harbour a mutation in the TGF-β type II receptor BMPR2 [32]. PAH shares some phenotypic features with aortic aneurysm syndromes since it is characterized by increased medial and adventitial thickness due to enhanced vascular smooth muscle cell or endothelial cell proliferation which can result in lumen loss [33].

## Conclusions

In conclusion, outgrowth endothelial cells may serve as a model to analyse alterations in gene expression in Loeys-Dietz syndrome. Gene expression profiling performed on LDS-OECs demonstrated pronounced up-regulation of bone morphogenic protein antagonist Gremlin-1 which may contribute to the vascular pathology of Loeys-Dietz syndrome. Furthermore, elevated Gremlin-1 plasma levels in LDS patients may serve as a new serological marker for early detection and diagnosis of Loeys-Dietz syndrome and as a potential follow up marker under a future targeted therapy.

## Supporting Information

File S1 **Supplemental methods (Flow cytometric analysis of endothelial cells) and supplemental tables S1 (Flow cytometric analysis of endothelial cells) and S2 (Primers and PCR conditions).**
(DOC)

File S2 **Supplemental table S3 (Expression values and permutation p-values).**
(XLS)
