# Supplementary material for: Overexpression of Gremlin-1 in Patients with Loeys-Dietz Syndrome: Implications on Pathophysiology and Early Disease Detection
Source: PLoS One. 2014 Aug 12;9(8):e104742. doi: 10.1371/journal.pone.0104742 (PMC4130545; doi:10.1371/journal.pone.0104742)
Supplement: File S1 — Supplemental methods (Flow cytometric analysis of endothelial cells) and supplemental tables S1 (Flow cytometric analysis of endothelial cells) and S2 (Primers and PCR conditions). (DOC) [file pone.0104742.s001.doc]

**File S1 - Supplemental Material**

**“Overexpression of Gremlin-1 in patients with Loeys-Dietz syndrome: Implications on pathophysiology and early disease detection “**

First author’s surname: Wellbrock

**Supplemental methods**

*Flow cytometric analysis of endothelial cells.*

OECs and HUVECs between passage 3-6 and 3-9, respectively, were analysed for expression of endothelial and haematopoietic markers by flow cytometry. Cells were harvested using trypsin, washed and resuspended in PBS + 10% human AB serum (Biotest, Dreieich, Germany). Cells were stained for 30 Min with fluorescein [isothiocyanate](http://en.wikipedia.org/wiki/Isothiocyanate)-labelled antibodies for CD31, CD146, CD45 and CD14 (BD Biosciences); with phycoerythrin-labelled antibodies for VEGFR1, 2 and 3 (all R&D Systems) and with an unlabelled CD144 antibody (BD Biosciences) followed by a second incubation with a secondary phycoerythrin-labelled anti-mouse IgG antibody. Cells were analysed using FACSCalibur and CellQuestPro Software (both BD Biosciences).

**Supplemental Tables**

**Supplemental table S**1. Flow cytometric analysis of endothelial cells.

|  | **Proportion of positive cells (%)*** | | | | | | | |
| --- | --- | --- | --- | --- | --- | --- | --- | --- |
| **Cell type** | **CD31** | **CD144** | **CD146** | **VEGFR-1** | **VEGFR-2** | **VEGFR-3** | **CD45** | **CD14** |
| **OEC (n=30)** | 92 ± 4 | 86 ± 6 | 94 ± 2 | 12 ± 6 | 50 ± 22 | 15 ± 11 | 1 ± 1 | 1 ± 2 |
| **LDS-OEC (n=3)** | 89 ± 8 | 87 ± 8 | 90 ± 7 | 3 ± 2 | 34 ± 17 | 16 ± 16 | 0 ± 0 | 0 ± 0 |
| **HUVEC (n=6)** | 97 ± 2 | 92 ± 4 | 97 ± 2 | 9 ± 7 | 25 ± 15 | 23 ± 16 | 0 ± 0 | 0 ± 0 |
| * mean ± standard deviation | | | | | | | | |

**Supplemental table S2. Primers and PCR conditions**.

| **Gene** | **Forward primer** | **Reverse primer** | **PCR product size** | **Annealing temperature** | **MgCl_2_** |
| --- | --- | --- | --- | --- | --- |
| ***GAPDH*** | gtcagtggtggacctgacct | tgctgtagccaaattcgttg | 245 | 61°C | 3 mM |
| ***GREM1*** | aacagtcgcaccatcatcaa | cgatggatatgcaacgacac | 220 | 61°C | 3 mM |
| ***BMP2*** | cccagcgtgaaaagagagac | ggaagcagcaacgctagaag | 222 | 60°C | 3 mM |
| ***BMP4*** | tctagcttgtctccccgatg | aaacttgctggaaaggctca | 214 | 60°C | 3 mM |
| ***BMPR1A*** | ggtttcatagcggcagacat | ctttccttgggtgccataaa | 198 | 59°C | 3 mM |
| ***LTBP1*** | tcctggggctttaacaaatg | cgatagctgcccatggtatt | 185 | 59°C | 3 mM |
